# Weather and Prey Predict Mammals’ Visitation to Water

**DOI:** 10.1371/journal.pone.0141355

**Published:** 2015-11-11

**Authors:** Grant Harris, James G. Sanderson, Jon Erz, Sarah E. Lehnen, Matthew J. Butler

**Affiliations:** 1 United States Fish and Wildlife Service, Albuquerque, New Mexico, United States of America; 2 Small Wild Cat Conservation Foundation, Corrales, New Mexico, United States of America; 3 United States Fish and Wildlife Service, Sevilleta National Wildlife Refuge, Socorro, New Mexico, United States of America; University of KwaZulu-Natal, SOUTH AFRICA

## Abstract

Throughout many arid lands of Africa, Australia and the United States, wildlife agencies provide water year-round for increasing game populations and enhancing biodiversity, despite concerns that water provisioning may favor species more dependent on water, increase predation, and reduce biodiversity. In part, understanding the effects of water provisioning requires identifying why and when animals visit water. Employing this information, by matching water provisioning with use by target species, could assist wildlife management objectives while mitigating unintended consequences of year-round watering regimes. Therefore, we examined if weather variables (maximum temperature, relative humidity [RH], vapor pressure deficit [VPD], long and short-term precipitation) and predator-prey relationships (i.e., prey presence) predicted water visitation by 9 mammals. We modeled visitation as recorded by trail cameras at Sevilleta National Wildlife Refuge, New Mexico, USA (June 2009 to September 2014) using generalized linear modeling. For 3 native ungulates, elk (*Cervus Canadensis*), mule deer (*Odocoileus hemionus*), and pronghorn (*Antilocapra americana*), less long-term precipitation and higher maximum temperatures increased visitation, including RH for mule deer. Less long-term precipitation and higher VPD increased oryx (*Oryx gazella*) and desert cottontail rabbits (*Sylvilagus audubonii*) visitation. Long-term precipitation, with RH or VPD, predicted visitation for black-tailed jackrabbits (*Lepus californicus*). Standardized model coefficients demonstrated that the amount of long-term precipitation influenced herbivore visitation most. Weather (especially maximum temperature) and prey (cottontails and jackrabbits) predicted bobcat (*Lynx rufus*) visitation. Mule deer visitation had the largest influence on coyote (*Canis latrans*) visitation. Puma (*Puma concolor*) visitation was solely predicted by prey visitation (elk, mule deer, oryx). Most ungulate visitation peaked during May and June. Coyote, elk and puma visitation was relatively consistent throughout the year. Within the diel-period, activity patterns for predators corresponded with prey. Year-round water management may favor species with consistent use throughout the year, and facilitate predation. Providing water only during periods of high use by target species may moderate unwanted biological costs.

## Introduction

Paradoxically, water is ubiquitous across many arid regions of Africa, Australia, and the United States [1–5]. Throughout these areas, wildlife management agencies established large numbers of water sources for wildlife since the mid 1900’s (e.g., via tanks, dams, bore holes, drinkers, troughs) [1–5]. The widespread assumption was that year-round water provisioning would bolster populations of herbivores (for viewing and harvest) and assist biodiversity [1–8]. Worryingly, these assumptions remain largely unfounded. The few studies in the southwestern United States indicate that water provisioning might assist populations of some species, though the results are equivocal [1,2,7,9]. Elsewhere, numerous projects from Africa and Australia demonstrate that water provisioning increases predator densities, predation on game, reduces biodiversity, and promotes range degradation [3,5,10–18]. Overall, there exists substantial evidence describing the direct and indirect negative effects of water provisioning on ecosystems, be it opposite results to those envisioned or unintended consequences. Studies demonstrating neutral effects of water provision, let alone beneficial ones, are sparse [1,2,9]. This situation casts doubts and fuels debates over the widespread practice of supplying water for wildlife [3–5,7,10,19,20].

Influenced by such research results, prominent natural areas like Kruger National Park (South Africa) closed many waterholes in the 1990’s to promote biodiversity and ecosystem processes [4]. Yet, despite the research results and the consequential management shifts occurring, the southwestern United States maintains a disconnect between providing water for wildlife and the biological ramifications it can have on biodiversity and ecosystem processes. Hence, water provisioning remains a popular management technique in the United States [1,2,19].

We hypothesized that differences in species’ water requirements (e.g., physiology), response to weather conditions, and predator-prey relationships would predict why and when mammals inhabiting the southwestern United States visited water. Our approach linked weather variables and the presence of prey with data from 36 remote cameras, which monitored the visitation to water for 9 mammals at Sevilleta National Wildlife Refuge (NWR), New Mexico, USA. Cameras operated from June 2009 through September 2014 and collected > 2.5M images. We focused our analyses on 4 ungulates (elk [*Cervus elaphus*], mule deer [*Odocoileus hemionus*], oryx [*Oryx gazella*], pronghorn [*Antilocapra americana*]), 2 lagomorphs (desert cottontail rabbit [*Sylvilagus audubonii*] and black-tailed jackrabbit [*Lepus californicus*]) and 3 carnivores (bobcat [*Lynx rufus*], coyote [*Canis latrans*] and puma [*Puma concolor*]).

As a first step, we ensured that sequential photos describing an animal’s visitation to water represented separate events. We then relied on generalized linear modeling to build associations between visitations, 5 weather variables and the presence of prey. The resulting models produce biological relationships explaining why these mammals visited water. Next, we used these models for making predictions that identify when (i.e., months) each species was most likely to visit water. Together, these model results and predictions provide ecological inference for advancing the understanding of relationships between weather, predators and prey in the southwestern United States. As a final step, we quantified the daily activity patterns for predators and their prey, to further examine the influence that water provisioning has on predator-prey relationships.

Our results provide data to help align the scheduling of water provisioning with the periods that target wildlife visit water most (typically harvestable or viewable game [2–5]). Doing so may serve to temper any unintended consequences that ensue from year-round water provisioning, such as increased predation, range degradation and biodiversity loss. Our objective centers on providing information to maximize the benefits of water management for target species while minimizing unwanted biological costs.

## Materials and Methods

### Study location

We conducted our study at Sevilleta National Wildlife Refuge (NWR), New Mexico, United States (latitude 34.30°, longitude -106.85°; 930.8 km^2^). The Refuge, bisected by the Rio Grande, is located at the junction of the following 4 ecoregions in New Mexico: the Arizona/New Mexico Plateau, the Arizona/New Mexico Mountains, the Southwestern Tablelands, and the Chihuahuan Deserts [21]. Characteristic flora of the area includes four-wing saltbush (*Atriplex canescens*), Indian ricegrass (*Oryzopsis hymenoides*), creosote bush (*Larrea tridentata*), black grama (*Bouteloua eriopoda*), and blue grama (*Bouteloua gracilis*; [22,23].

### Trail camera deployment

Cameras were located at 7 natural and 29 artificial sources of water. Natural water sources consisted of springs and artificial sources included tanks, tractor tires, tubs or troughs filled by solar powered pumps. All locations provided water consistently throughout this study, and we analyzed both types of water sources together.

We used Reconyx (model HC550; http://www.Reconyx.com) and Bushnell (model 119435; http://www.Bushnell.com) passive infrared triggered cameras to monitor water use. We deployed cameras on a minimum of 23 and a maximum of 36 water sources, from June 29, 2009 through September 15, 2014 (1,905 days; 53,864 “camera trap days”). Once set, cameras remained stationary. Elevation at camera locations ranged 1,469 to 1,985 m, and the area of the convex polygon sampled by all cameras was 867 km^2^ (S1 Table). The average distance between each camera and its closest neighboring camera was 1.6 km (SD = 2.3). Cameras were set with at least a 10-sec delay occurring between triggers. All pictures were relabeled, stored, and analyzed using the methodology and computer programs described in these citations [24,25]. The United States Fish and Wildlife Service provided permission for this study and approved all sampling procedures.

### Temporal resolution

Often, species visitations to water are captured with multiple images. For our analysis, we identified independent visits to water, for each species. In this context, independence meant identifying the amount of time elapsing between sequential images that constituted a new visit. We started by assuming that if at least one hour elapsed between images then two independent visits occurred. This allowed us to estimate the duration of a visit and the time between visits for the 9 species (i.e., the time between two independent visits, by the same or different individuals of the same species; “return time”). In other words, return time represents the following: once an individual or group of the same species finished visiting a site, the amount of elapsed time before that species (singly or in a group) was recorded again, at that same site. The hour interval is arbitrary, though it can be any amount of time provided it exceeds the actual duration that a species visits water (see Discussion).

For example, imagine a series of 6 bobcat images taken at the following times (sequentially, from the first image): 0, 10, 40,160, 3880, and 3900 sec. The amount of time elapsed between images is 10, 30, 120, 3720, and 20 sec. According to our method, this would generate 2 independent visits. The duration of the first independent visit was 160 sec and the second lasted 20 sec. The return time was 1 hour and 2 minutes. Using this approach, we calculated the duration of independent visitation events and the return time for the 9 species, at each camera, during the study period.

For all species, the 99^th^ percentile of visit durations were <51 min and the mean return time >20 hours (see Results). Therefore, we considered sets of images of the same species separated by an hour or more to represent different visits (i.e., independent sets of images). For each species, we then identified the maximum number of individuals recorded in a single image within each set of independent visits, at each site. We summed these counts within a weekly period (separately for each species across all sites). For example, if cameras recorded 3 independent visits for a species during a week in which the maximum number of individuals recorded during each visit was 4, 10, and 3, we assumed that the species visited water 17 times during that week. This total count indexed the number of visits made to water for each species during each week, and formed the response variable in the analyses (hereafter, visitation).

### Predictor variables

We modeled visitation to water for 9 different mammal species: black-tailed jackrabbit, bobcat, coyote, desert cottontail rabbit, elk, mule deer, oryx, pronghorn, and puma as a function of weather variables. Weather data were collected every 10 mins, and originated from 5 weather stations operated by the Long Term Ecological Research Station (LTER; http://www.lternet.edu) based at Sevilleta NWR. We pooled weather variables across the stations as weekly means to match the resolution of visitation. The weather variables included maximum temperature (TMAX), relative humidity (RH), vapor pressure deficit (VPD), and precipitation. We included mean maximum temperature (TMAX) because hotter temperatures increase moisture stress on plants and animals, which may cause animals to visit surface water more frequently [26,27]. Relative humidity (RH) describes the amount of moisture content in air. For each week, we averaged the minimum RH value from each day, since animals and plants lose water faster during periods with low RH [27]. Vapor pressure deficit (VPD), which is the difference between actual and saturated water vapor pressure, is a metric describing evapotranspiration. Elevated VPD pulls moisture from animals and vegetation, and for the latter, increases desiccation rates and reduces digestibility [27–29]. For these reasons, predators and herbivores may seek surface water as VPD increases [30]. We calculated VPD at a resolution of 10-min intervals (http://biomet.ucdavis.edu/biomet/VPD/vpd.htm) as

$$VPD\;=\;e^{\left(\frac{17.27*TMAX}{TMAX+265.5}\;\right)*\left(0.6108-0.006108*RH\right)}$$

For each week, we averaged the maximum VPD calculated for each day.

Precipitation may influence animals’ visitation to permanent water sources for two reasons. First, rainfall can fill temporary pools and enhance flows from seeps and springs. This may increase the availability of surface water, so animals visit permanent water sources less. Second, rainfall increases water content in plants, allowing ungulates to meet their water requirements from forage [27,30]. Therefore, we incorporated the timing and amount of precipitation in our models. For example, the current week may have low precipitation, but were precipitation also low over the prior 4 weeks, a mammal may need to visit water more frequently. We examined this temporal aspect (e.g., long-term precipitation) by estimating the total amount of precipitation for 1 to 9 weeks prior to visitation. Thus, we created 10 precipitation variables (i.e., 1-week precipitation total to 10-week cumulative precipitation total). We examined the Pearson’s correlation coefficient between each of these precipitation variables and 1-week precipitation. The total amount of precipitation over the current week and the prior 5 weeks (i.e., a 6-week precipitation total) was the first weekly cumulative total to be uncorrelated with the 1-week total (r = 0.49). Therefore, we included the weekly total of precipitation (P1) and the total 6-week precipitation (hereafter “long-term precipitation”; P6) as separate variables. We examined the temporal aspect for the other weather variables but found all long-term averages, up to 10 weeks prior, were correlated with the 1-week average.

For predator species, we modeled their visitation to water as a function of prey visitation (count based). For puma, prey consisted of oryx, elk, mule deer and pronghorn. Bobcat prey was cottontail rabbits and jackrabbits. Coyote prey included all the ungulates and lagomorphs.

We examined the Pearson’s correlation coefficient between the weather variables (TMAX, RH, VPD, P1 and P6) and each species, to ensure we did not induce multicolinearity in our models (S2 Table). Therefore, we did not allow TMAX and VPD or RH and VPD to occur together in models (r > 0.55).

Our process to identify species captured by the imagery did not differentiate between young and adults. Therefore, we included week number (i.e., spanning 1–52) as a quadratic variable to account for the possibility that increased visitation to water stemmed from a birthing pulse.

### Modeling steps

We built predictive models using data from 2009–2013. Data from 2014 was reserved to evaluate model fit. We modeled relative frequency of visitation using generalized linear models based on the MASS package [31] in program R [32]. We assumed a negative binomial distribution, which allowed us to accommodate overdispersion of the counts [33]. The log of the number of camera trap days operational during each week served as a linear offset, which transformed the response to daily visitation per week [33]. Models contained each explanatory variable singly, or in multiple combinations with first order interactions, provided the variables were uncorrelated.

We evaluated 93 competing models for each herbivore, and approximately 150 models for each predator (number of models varied by inclusion of different prey species, alone or in combination). The best models were selected using Akaike’s information criterion (AIC; [34]). We ensured that final models had informative and significant parameters [35] and lacked heteroskedasity and multicollinearity (based on variance inflation factors). We evaluated the fit of the final models by using pseudo R^2^ (based on deviance residuals; [33,36]) and by plotting the model predictions against the observed values. We also standardized each variable (i.e., (value–mean)/SD) in the best models to evaluate which variable(s) influenced visitation most.

We identified seasonal patterns of visitation for each species, by examining the prediction of visitation based on weekly averages of each weather variable across 2009–2013. To help interpret the results for each species, we divided these predictions by the maximum visitation value, to convert the predictions into proportions. We considered predicted proportions ≥66.66% indicative of when species visited water most, and proportions ≤ 33.33% when species visited water least. We also used the weather data from 2014 to predict water visitation for 2014. We compared the 2014 results against the results from the 2009–2013 data to evaluate the predictive ability of each model. For predators, we filled in the few months of 2014 lacking prey data with the results of the predictions for their respective prey.

Lastly, we examined daily activity patterns for each species. Within each hourly period we determined if a species was present or not for every camera and day. We then counted the number of presences recorded, within each hourly period, across all cameras (generating a frequency of activity for every hourly period). We converted the frequency of activity for each hourly period into a proportion, by dividing by the total number of times a species was recorded present throughout the study.

## Results

Combined, all cameras recorded 2,577,231 images. Of these, 1,968,340 images captured 9 species of mammals (Table 1). The most common species included mule deer, pronghorn, and elk (Table 1).

**Table 1:** Summary Statistics for Camera Imagery with Visit Durations and Species Return Times at Water Sources.

				Duration (min)		Return time (hr)	
Species	# Images	# Independent	% Reduction	50^th^ %	99^th^ %^a^	Mean	SD
Mule Deer	952,659	31,861	96.7	5	32	24.2	0.8
Pronghorn	581,163	14,654	97.5	4	45	20.6	1.6
Elk	237,615	6,308	97.4	5	39	70.9	5.2
Oryx	108,762	2,929	97.3	4	51	156.2	12.3
Coyote	61,881	11,178	81.9	1	10	89.8	3.4
Jackrabbit	13,041	3,177	75.6	1	8	200.8	13.8
Cottontail	7,580	2,236	70.5	1	6	309.3	23.2
Puma	3,625	587	83.8	1	12	884.0	95.9
Bobcat	2,014	665	67.0	1	6	764.3	69.5

The total number of images, number of independent images, and the amount of reduction in data by analyzing independent images to predict visitation to water for 9 mammals inhabiting Sevilleta National Wildlife Refuge (New Mexico, USA). The table includes the duration of visits to water which represents the amount of time spent visiting water. Return time indicates the average time between independent visits at a site.

^a^ The duration that < 99% of all visits lasted (minutes)

We quantified the duration of time each species visited water at a site, and the return time for these 9 mammals. Carnivores and lagomorphs had the shortest duration of visits and ungulates had the longest (Table 1). Median duration for all species was ≤ 5 min and 99% of all visit durations were ≤ 51 min (Table 1). Both felids had the longest return time to a site (>1 month). Of the ungulates, oryx had the longest return time to a site (6.5 days) while pronghorn had the shortest return time (< 1 day; Table 1). Cottontail rabbit and jackrabbit had return times to a site of approximately 13 and 8 days, respectively (Table 1).

### Models of water visitation

**Fig 1:**
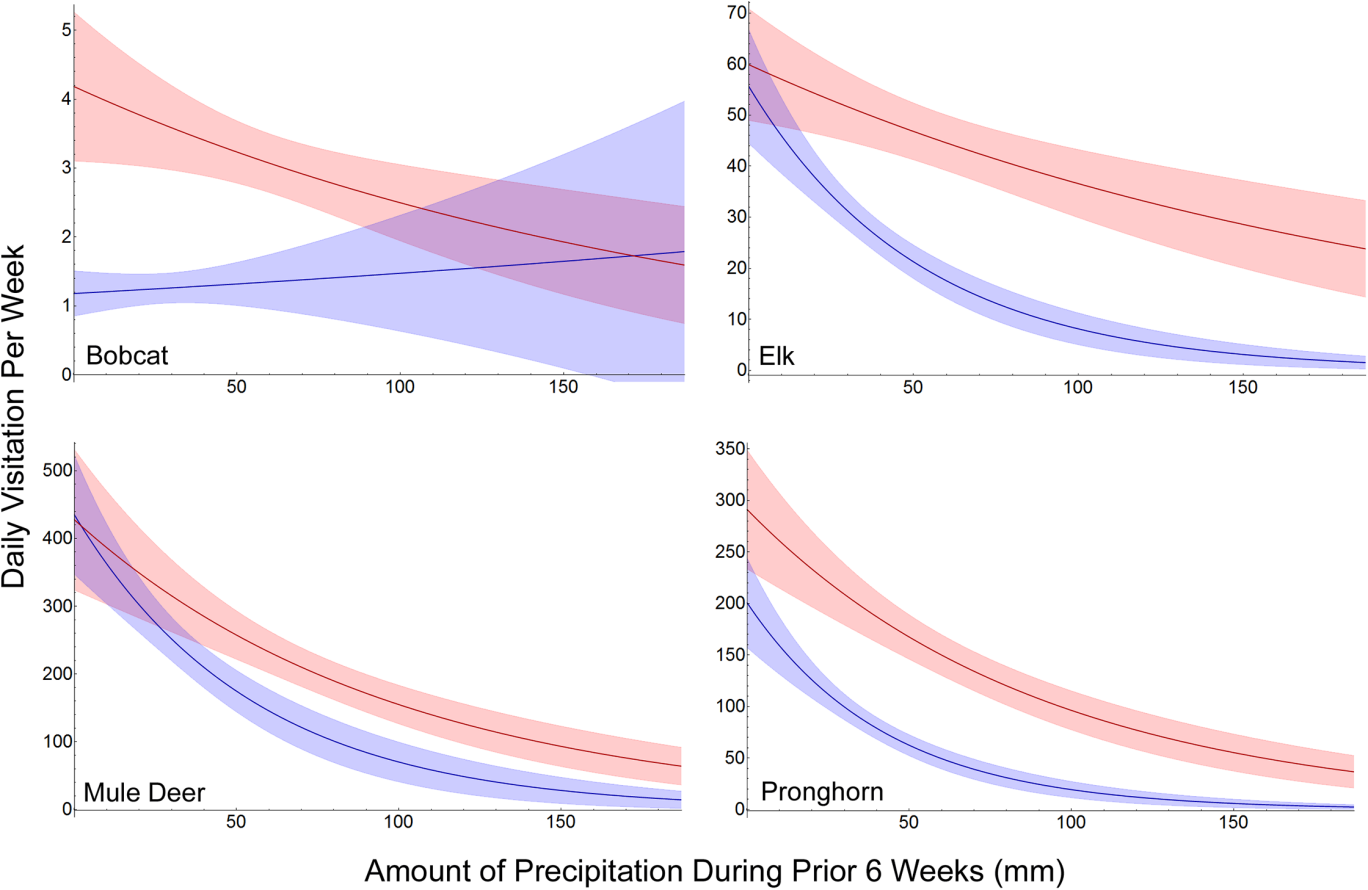
Variable Interaction. Interaction between maximum temperature (weekly average) and long-term precipitation (6-week total) in the models receiving most support (lowest AIC) for predicting visitation to water for bobcat, elk, mule deer and pronghorn inhabiting Sevilleta National Wildlife Refuge, New Mexico, USA. Blue indicates the model prediction with maximum temperature held at the lower quartile. Red indicates the model prediction with maximum temperature held at the upper quartile. Shading colors the 95% confidence interval for the predictions describing the amount of visitation to water.

**Fig 2:**
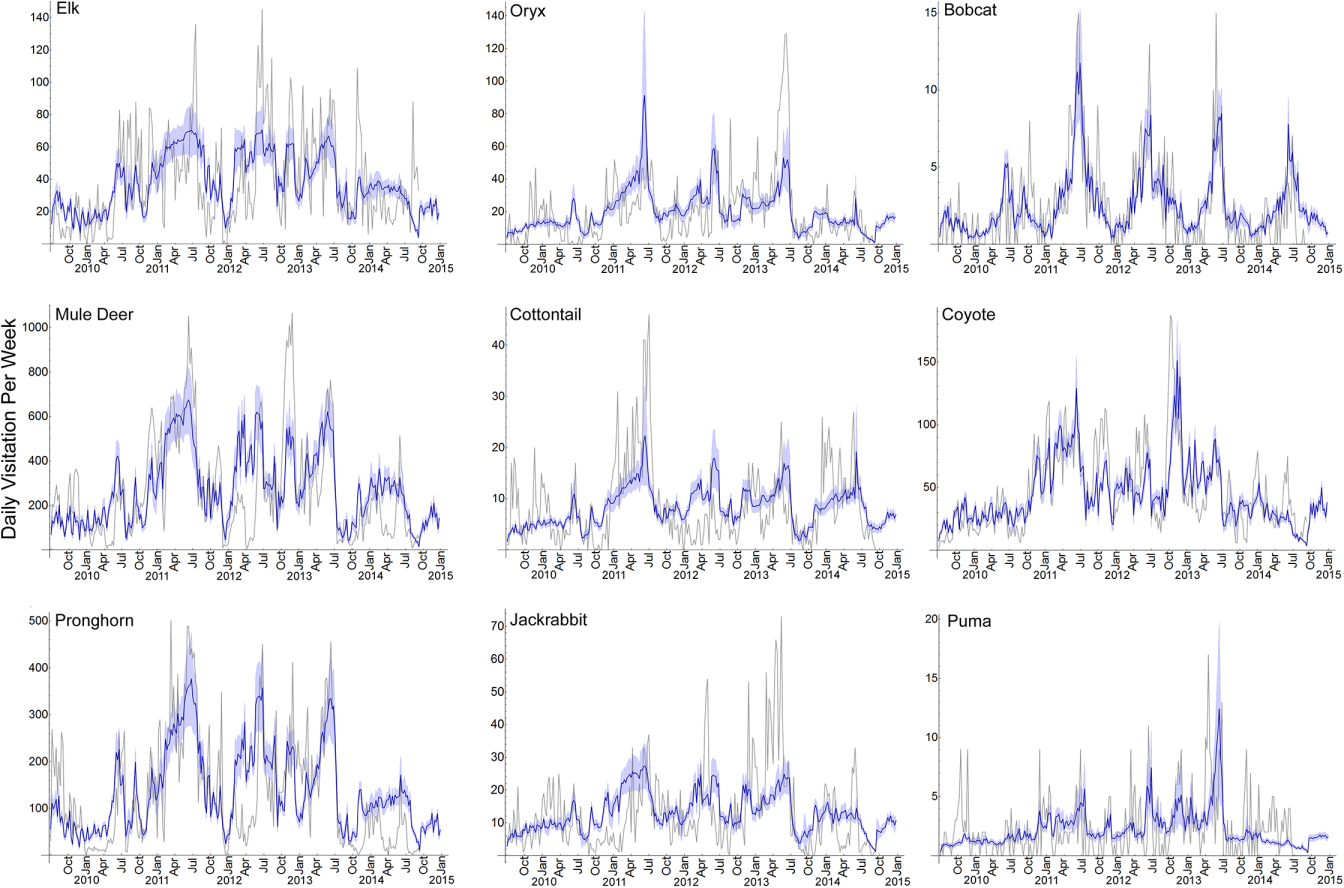
Model Fit. Visualization of the best model fit predicting the total visitation to water per week (blue line, with 95% confidence intervals as light blue coloring) for 9 mammals inhabiting Sevilleta National Wildlife Refuge, New Mexico, USA. Model predictions span June 29, 2009 through December 31, 2013, with data from 2009–2013 used to fit the model. Model projections occur from January 1, 2014–December 31, 2014. Gray line indicates the actual number of visits observed each week.

Visitation to water by the 3 native ungulates, elk, mule deer and pronghorn, was best described with maximum temperature, long-term precipitation (i.e. 6-week precipitation), and an interaction between them (Table 2; Fig 1). Visitation decreased as precipitation increased (Table 2). Warmer temperatures generally increased visitation, but when precipitation was minimal, visitation was similar irrespective of temperature (Fig 1). The standardized model coefficients indicated that long-term precipitation had a stronger influence on visitation than maximum temperature for these ungulates: 22% more for elk, 3 times more for mule deer, and 33% more for pronghorn. The mule deer model included RH (Table 2), and long-term precipitation had more influence than it (2.5 times more). These models fit well and were consistent with the underlying data (Table 2; Fig 2).

**Table 2:** Herbivore Visitation.

Model Variable^a^	Elk	Mule Deer	Pronghorn	Oryx	Cottontail	Jackrabbit 1	Jackrabbit 2
AIC	2096.00	2976.03	2668.81	1843.60	1411.86	1642.09	1643.20
# Parameters^b^	5	6	5	5	4	5	4
AIC weight	1.00	0.73	1.00	0.51	0.69	0.35	0.20
Pseudo R^2^ (%)	23.59	38.10	36.14	21.63	19.46	16.85	15.81
Intercept (SE)	-1.237 (0.177)	1.235 (0.256)	0.127 (0.191)	-1.775 (0.120)	-2.864 (0.108)	-1.871 (0.161)	-2.334 (0.108)
Precip6 (SE)	-0.029 (0.005)	-0.024 (0.005)	-0.031(0.005)	-0.006 (0.003)	-0.010 (0.002)	-0.015 (0.004)	-0.010 (0.001)
VPD (SE)				0.017 (0.005)	0.009 (0.004)		0.007 (0.004)
TMAX (SE)	0.004 (0.008)	-0.001 (0.009)	0.021 (0.008)				
RH		-0.018 (0.006)				-0.018 (0.007)	
TMAX:Precip6 (SE)	8.3e-04 (1.8e-04)	4.7e-04 (1.7e-04)	7.1e-04 (1.9e-04)				
VPD:Precip6 (SE)				-3.6e-04 (2.0e-04)			
RH:Precip6 (SE)						2.2e-04 (1.3e-04)	

Models of visitation to water for 4 ungulates and 2 lagomorphs inhabiting Sevilleta National Wildlife Refuge, New Mexico, USA.

^a^ Table includes models with informative parameters and ΔAIC < 2.0. Table excludes one oryx model and 2 jackrabbit models that were subsets of the models presented (S3 Table). Model coefficients are not standardized.

^b^ Number of parameters includes the intercept and dispersion parameter.

The best models for oryx and cottontail rabbits indicated that visitation increased as VPD increased and long-term precipitation decreased (Table 2). Long-term precipitation had more influence than VPD on visitation: 15 times more for oryx and 3 times more for cottontail rabbits. These models fit well (Table 2) and were consistent with the underlying data (Fig 2).

For jackrabbits, increased precipitation and RH reduced visitation, while increased VPD raised visitation. Long-term precipitation had most influence on the models, 4 times more than RH. These models fit worse than the other herbivores (Table 2), though the patterns of the prediction matched the data observed (Fig 2).

Precipitation, RH, maximum temperature and the presence of prey predicted visitation by bobcats and coyotes best (Table 3). As RH increased, visitation declined for bobcats and coyotes, while increased weekly precipitation also reduced coyote visitation (Table 3). For bobcats, visitations declined with increased long-term precipitation, though the interaction between temperature and precipitation neutralized this relationship at low temperatures (Fig 1).

Bobcat visitation was also concurrent with visitation by cottontail rabbits or both cottontails and jackrabbits, whereas coyote visitation was concordant with mule deer visitation (Table 3; S3 Table). The standardized model coefficients indicated that for bobcats, maximum temperature was 6.5 times more influential than prey. Alternatively, for coyote, mule deer was 1.4 times more influential than temperature. Long-term precipitation had weaker predictive ability, and was 1.6 times less influential than prey for bobcats and 3.6 times less than prey for coyote.

**Table 3:** Predator Visitation.

Model Variable^a^	Bobcat	Coyote	Puma
AIC	834.98	2035.96	914.82
# Parameters^b^	7	8	5
Model Weight	0.22	0.55	0.49
Model Pseudo R^2^ (%)	45.58	42.58	16.09
Intercept (SE)	-5.452 (0.310)	-1.081 (0.172)	-4.947 (0.125)
Precip1		-0.021 (0.007)	
Precip6	0.007 (0.006)	0.001 (0.001)	
RH (SE)	-0.018 (0.007)	-0.017 (0.004)	
TMAX	0.073 (0.010)	-0.020 (0.005)	
TMAX:Precip6	-4.3e-04 (2.3e-04)		
Precip1:Precip6		1.4e-04 (7.1e-05)	
Cottontail (SE)			
Lagomorphs (SE)^c^	0.005 (0.003)		
Mule deer (SE)		0.001 (1.6e-04)	5.1e-04 (3.5e-04)
Elk (SE)			0.006(0.003)
Oryx (SE)			0.011(0.003)

Models of visitation to water for bobcat, coyote and puma inhabiting Sevilleta National Wildlife Refuge, New Mexico, USA.

^a^ Table includes models with informative parameters and ΔAIC > 2.0. Table excludes three bobcat models, one coyote model and one puma model that were subsets of the models presented (S3 Table). Model coefficients are not standardized.

^b^ Number of parameters includes the intercept and dispersion parameter.

^c^ Lagomorphs consisted of cottontail and jackrabbit visitation combined.

For puma, visitation was best predicted by the presence of elk, mule deer and oryx (Table 3). Based on the standardized model coefficients, oryx had 1.4 times more predictive power than elk and 2 times more than mule deer. The puma models fit poorly (low Pseudo R^2^ and prediction did not track observed data well; Fig 2). All models within 7 ΔAIC of the model receiving most support are included in a supplementary file (S3 Table).

Since the time variable was correlated with temperature and VPD, it may appear that results based on the weather variables could indicate a timing effect, such as a birthing pulse, instead of weather factors. This notion is unsupported, since no species had a competing model include time (alone or in combination with other variables [Tables 2 and 3; S3 Table]). Therefore, weather variables were superior predictors.

### Seasonal visitation patterns around waterholes

**Fig 3:**
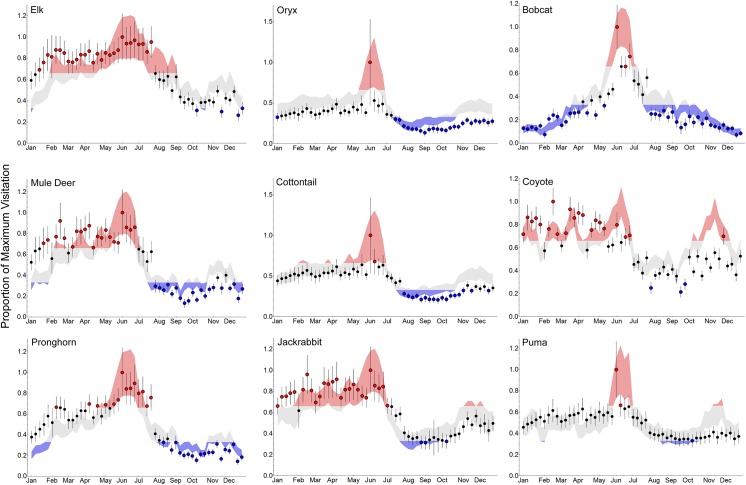
Monthly Timing of Visitation. Timing of visitation to water for 9 mammals inhabiting Sevilleta National Wildlife Refuge, New Mexico, USA. The shaded area encompasses the prediction and confidence interval from the best model for weekly visitation, using weather averages during the modeling period from June 29, 2009 through December 31, 2013 (and visitation by prey for carnivores). The points represent model predictions using weather data and prey visitations (as appropriate) from 2014. All predictions were divided by the maximum visitation value, to generate a proportion. Red colors values > 66.66% (high use) and blue points colors values < 33.33% (low use). Black lines on the points indicate confidence intervals. Predictions from 2014 corresponded with model predictions from 2009–2013.

Linking visitation with weather enabled us to identify when each species visited water during the year. We built the model predictions based on data from 2009–2013, and used data from 2014 to evaluate them (Fig 3). The 2014 predictions correspond with those based on 2009–2013, indicating good predictive ability (Fig 3).

All four ungulates exhibited peak use of water during May and June (Fig 3). Oryx had the shortest duration of peak use (2 months), while elk had the longest peak use from January through August (8 months). Peak visitation for mule deer spanned 5 months (February through June), while peak use for pronghorn spanned 3 months (May through July).

Coyote visitation peaked during 8 months (January–June and October–November), and puma visitation peaked in May and June. Both predators lacked a clear period when visitation was below a third of maximum visitation (Fig 3). Bobcats had the most variation in seasonal use with a peak spanning 2 months (May and June), with low visitation from July through April.

### Daily activity patterns

**Fig 4:**
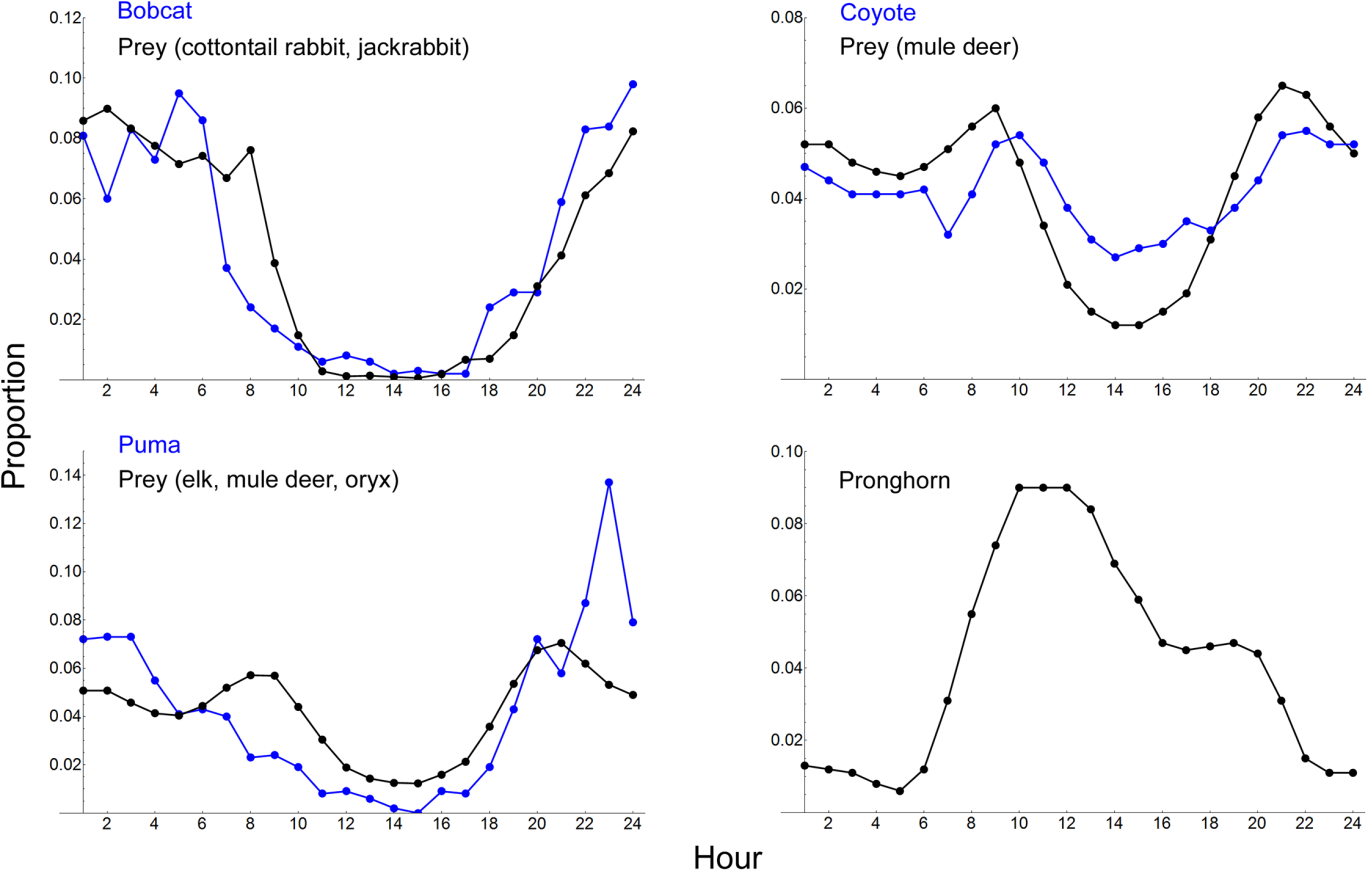
Activity Patterns. Daily activity patterns (based on species presence/absence at an hourly resolution) for mammalian predators (blue) and prey (black) at Sevilleta National Wildlife Refuge, New Mexico, USA, from June 29, 2009–September 15, 2014. Prey was identified based on the models receiving most support, which predicted visitation to water for each predator. None of the predator models included pronghorn as a predictor for water visitation. The vertical axis represents the proportion of visitation.

Predator visitation tracked the daily patterns of prey visitation to water (Fig 4). Bobcat diel activity was predominately nocturnal, along with cottontail rabbits and jackrabbits. Mule deer diel activity peaked during crepuscular hours, with a large portion of visitation occurring at night. Coyote matched this pattern, though the differences between nighttime and daytime visitations were less pronounced. Puma diel activity tracked prey (elk, mule deer and oryx) with most visitation during nocturnal and crepuscular hours. In contrast to the other species, pronghorn diel activity peaked at midday, with little visitation occurring nocturnally (Fig 4).

## Discussion

Wildlife agencies in Africa, Australia and the United States established programs to provide surface water for wildlife over half a century ago [1–10]. Water provisioning was designed to increase the amount of range available to ungulates during dry seasons (due to water scarcity within dry season ranges), and help mitigate the effects of anthropogenic barriers and human exploitations, that prevented animals from accessing surface water [1–5,7,37]. Increased populations of herbivores and enhanced biodiversity were the intended results.

Most research describing the effects of water provisioning on wildlife abundance, biodiversity and ecosystem health comes from studies in Australia and Africa. Elsewhere, notably the United States, research examining such relationships remains limited [1,2,7,9].

We addressed this topic by establishing a simple monitoring design to determine why species visited water and quantify when they do. We focused on mammals, examining associations between visitation, weather, and prey. Our results advance understanding the factors underpinning species visitations to water, and the effects that managed waters have on interspecific relationships. Indeed, understanding the effects of water provisioning in arid ecosystems has important implications for sustainably managing predator-prey systems (see below; 10,15).

### Seasonal visitation patterns around waterholes

Access to surface water constrains herbivore movements and alters their distributions during dry seasons [38–40]. In our study, all 6 herbivores visited water most frequently during the driest period this area experiences (high temperatures, low RH, and low precipitation), occurring from May through June (Fig 3; [23]). Given that long-term precipitation affected herbivore visitation most, the direct effects of weather were likely on the water content of forage [30,41–43]. In July, monsoons bring geographically patchy bouts of rain that fill temporary pools and recharge soil moisture, producing flushes of new vegetative growth [23]. Animals can then obtain water outside of permanent areas, either in forage or freestanding from temporary pools.

The influence of arid conditions on species dependence for surface water is largely contingent on their foraging strategy. In African savannas, grazing herbivores are typically more dependent on surface water, with browsers less so [40,41]. The mechanism lies with grasses having less moisture content than browse during the dry seasons, such that grazers obtain inadequate moisture from forage [30,42,43]. The differences in water requirements for grazers and browsers were inferred by the 6 herbivores in our study. For instance, elk, despite their dietary plasticity between grasses and browse, are predominantly grazers [44,45]. Visitation to permanent water dropped precipitously for all of these ungulates, except elk, once monsoon season began. Elk also had the longest period of water use of all the herbivores analyzed, and no clear period when they did not visit water (Fig 3).

Oryx, native to extremely arid regions of southern Africa, were introduced to New Mexico (USA) in 1969 [46]). In New Mexico, oryx consume large amounts of shrubs and forbs, a diet differing from those inhabiting southern Africa, which are predominantly grazers [47]. In both locations, oryx have considerable physiological adaptations to conserve water including selective feeding, voluntary hyperthermia, and lowering metabolic rate [42,48–50]. Such factors–sizeable browse diet and extensive physiological adaptations–help explain why oryx displayed the shortest period of water visitation in New Mexico.

Mule deer diets consist primarily of browse, and pronghorn diets are mostly shrubs and forbs [51,52]. Mule deer visited water over a longer period than pronghorn, implying a greater reliance on surface water, though less than elk. Pronghorn visitation was more similar to the patterns displayed by oryx (Fig 3).

Grasses and forbs are the predominant forage for cottontail [53], while jackrabbit diets focus on shrubs and forbs [54]. Jackrabbits visited water more often throughout the year than cottontails (Fig 3). This may stem from cottontails having behavioral and physiological adaptations, enabling them to survive drought by ingesting cactus and forbs with high moisture contents [53]. Granted, jackrabbits have considerable (and similar) physiological adaptations for surviving drought too [55]. Like the other herbivores, cottontails and jackrabbits visited surface water or succulent forage in proximity to water during the most arid periods [Fig 3; 56,57].

Bobcat visitation increased with visitation by cottontails and jackrabbits (Table 3). Visitation by mule deer had the greatest influence on visitation by coyote, and prey solely predicted puma visitation (elk, mule deer, and oryx). The activity patterns for predators also resemble the activity patterns of their prey (Fig 4). These results are sensible, as bobcats consume both lagomorphs [58,59], numerous studies report that coyotes predate adult deer throughout the western United States [e.g., 60,61], and in North America, large prey forms the primary diet of puma [62,63].

We interpret the associations between visitation to water by predators and their prey as predators visiting water to increase hunting opportunities. Granted, these patterns may demonstrate behavioral and physiological commonalities between the species, instead of predation. We explored this notion by incorporating the visitation of each predator into the model receiving most support for each herbivore. Visitation by puma improved model fit for elk, oryx and mule deer, although the variable describing long-term precipitation remained the most influential (3 times more; based on standardized variables). For deer and pronghorn, the addition of coyote improved model fits, with the variable describing long-term precipitation being 3 times more influential for deer and 6 times more influential for pronghorn. The addition of predator visitation did not improve model fits for cottontail rabbits or jackrabbits.

Therefore, the explanations describing why prey visit water centers on low amounts of long-term precipitation. Understandably, when prey visit water, the predator may visit too, because the predators are hunting the prey. Hence, visitation by the predator may improve model fits for the prey, only because the predators track them, making the relationships between prey and predator harder to separate. As such, the associations between predator and prey in our models are best explained by increasing hunting opportunities for the predators, and less so from commonalities in behavior or physiologies between species.

That predators frequent artificial water to hunt is not unique to the southwestern USA. In Africa and Australia, artificial waterholes form passive traps for prey, making them centers for predation [3,15]. For example, at Hwange National Park, Zimbabwe, water provision drove lion (*Panthera leo*) movements, habitat selection and hunting behavior [15–17]. Lions preferred making kills within 2 km of a waterhole during all seasons [15]. In the Serengeti, Kruger National Park and Klaserie Private Nature Reserve, water provision also attracts ungulates and therefore influences lion habitat selection [64–66]. In the Serengeti, lions favored areas where prey was easy to capture–locations with freestanding water–over locations with higher prey density [67]. In Kruger National Park, the introduction of water encouraged zebra (*Equus quagga*) and blue wildebeest (*Connochaetes taurinus*) to inhabit areas they historically did not. Lions followed this influx, and their predation contributed to a 10-fold decline in rarer antelope (roan antelope; *Hippotragus equinus*) inhabiting this area [65]. At Klaserie Private Nature Reserve, water was the main factor determining the kill location for lions, with kills closer to managed waters than natural river sources [66]. In Australia, introduced feral cats (*Felis catus*), foxes (*Vulpes vulpes*) and dingos (*Canis lupus*) commonly occur at artificial water points [3]. Predation by fox and feral cats caused large declines in Australian fauna [68]. Dingos frequent watering points for hunting opportunities, and act as the main regulator of kangaroo populations in eastern Australia [69]. Water provision is implicated in the range expansion and population increases of these predators [3,18].

Given this information, it becomes clear that the number and spacing of artificial sources of water across arid ecosystems changes the distribution patterns of species [3,40,41]. Water provision enables species more reliant on surface water to expand into locations that were previously uninhabitable [3,40,65]. These outcomes can negatively affect conspecifics [3–5,65].

In our study, elk, coyote and puma had few periods when visitation declined below 33.33% of maximum, suggesting that elk, coyote and puma visit water evenly, throughout the year (Fig 3). In this location, these species may be the main beneficiaries of a year-round watering regime. As shown above, water provisioning may enable these species to use areas that they historically would avoid (or persisted in at lower numbers), which may negatively affect species more adapted to arid conditions that use these areas, thereby exacerbating declines in their populations or increasing predation rates [5,9,65,67,70].

### Model fits and image independence

Generally, the predictions of species visitation matched the observed patterns, especially for the ungulates. Exact matches are unexpected, because even during arid conditions, animals will not always visit surface water. Visiting permanent sources of water can be dangerous and physiologically taxing, as animals must remain alert for predators and overcome any intra-specific social issues occurring at water [30,48,71]. Therefore, animals have strategies such as physiological changes to reduce water loss, behavioral responses (e.g., seeking shade, reducing movement, group formation, heightened vigilance), and selective feeding to maximize the ingestion of pre-formed water, to avoid visiting permanent sources of water [30,50,71,72]. Movement by animals on and off the study area, visits to unmonitored water sources, extremes in weather not effectively captured by the weekly means, and spatial variation in precipitation and other weather events may have also added unmodeled variation to the data.

To calculate duration and return time of species visitation to water, we used one hour to indicate independent visits. This time could be set to any value, provided the value exceeds the duration time. As our calculations show, for all species, the duration of a visit was below one hour, and the return time substantially above one hour (Table 1). Therefore, we considered sets of images of the same species separated by an hour to represent different visits (i.e., independent sets of images).

Using one hour to separate independent visits reduced the number of pictures used in analyses by > 95% for the ungulates, ~80% for coyote and puma, and ~70% for the lagomorphs and bobcats (Table 1). With many pictures unused, it implies that cameras could operate with a coarser setting to capture images (i.e., >10 sec. trigger delay), thereby reducing the amount of data to process. This outcome could lessen the data management burden placed on many projects relying on imagery from remote cameras.

### Water provision and species management

Given our results and the above information, we can evaluate the justifications for water provision, and suggest improvements on how to manage water for these species. Although the common objectives of water provision–increasing ungulate populations and assisting biodiversity–are not mutually exclusive, we handled each in turn.

Visitation to water is predictable for these 9 species (Tables 2 and 3). Therefore, the potential exists for biologists to manipulate water by providing it when the target mammal(s) use it most, and removing water when non-target animals use it more (providing these periods differ). In the southwestern United States, the target mammals typically include harvestable game, such as elk, mule deer, pronghorn and oryx [1,2,7]. If managed waters are established for these ungulates, then they would likely benefit most by having water available during their peak use, May and June, which could be expanded to February through July, to better accommodate elk. Because these periods are defined mainly by precipitation, they could be adjusted based on the onset of monsoons in July, or the amount of spring rainfall received. An additional, ecological benefit of providing water only during certain periods of the year instead of year-round is lowering pressure on water resources (i.e. pumped groundwater). Therefore, providing water during periods of high use by target species promotes water conservation in arid environments.

Presumably, management that provides water when these ungulates visit water less (August through January) has lower value for these species. Therefore, in New Mexico, instead of providing water year-round, anthropogenic water sources could become unavailable during this period of low use. This action may discourage puma and coyote from frequenting these areas, during such periods. This outcome is likely, given that water provisioning can enable predators to inhabit areas previously unhospitable [5,10,65]. A result of having water unavailable year round may be a reduction of predation on game. This outcome is also plausible, given that locations with provided water tend to enhance predation, year-round [3,10,15–17]. If these informed hypotheses are valid in the United States, then seasonal water provisioning could form a management alternative to assist game while reducing predator control (because fewer predators may be present, along with fewer predation events). Clearly, more work is required to evaluate these ideas.

This discussion brings in the second justification for watering desert fauna: assisting biodiversity, which assumes a benefit to all species [1,6]. Our work with 9 mammals shows that some species visit water over longer periods than others do. This outcome demonstrates that having water available year-round benefits species unequally, which could catalyze biodiversity loss. We explain with a related example. Desert grasslands are nitrogen limited [73,74], so it might be logical to conclude that supplementing them with nitrogen will increase their species diversity. Instead, supplementing arid grasslands with nitrogen lowers species diversity [75–77] and reduces grassland productivity [73]. Reason being, grasslands contain plants with differing abilities to incorporate nitrogen. While all the species seem to benefit from increased nitrogen, over time nitrophilic plants competitively exclude the other species adapted to less fertile conditions [75,77]. This leads to changes in community composition favoring nitrophilic plants, and biodiversity loss.

Relatedly, are the direct and indirect effects of establishing water on range condition. Historically (the unmodified condition), large herbivores inhabiting arid areas frequently moved (nomadically or migrations) to locations having sufficient water and forage, given the few sources of permanent water available [3,78–80]. This phenomenon generated patterns of light grazing interspersed with shorter periods of intense grazing [81]. Water provision usually results in the herbivore populations becoming sedentary [10,82], thereby changing their distributions and patterns of landscape use [3,40]. During dry seasons, herbivores aggregate near the water, generating heavy grazing pressure that degrades the range condition by reducing the composition of herbaceous vegetation, fine fuels, basal cover, and standing crop [13,14,81,83–85]. The persistence of grazing pressure increases unpalatable perennial shrubs, reduces palatable native perennial grasses, and causes declines in the species diversity of habitats [3,12–14]. While the severest effects on herbaceous vegetation occur within ~200–500m [3,85], the effects can extend farther than 10 km. Hence, places with distances between permanent sources of water < 10 km typically lack grazing relief [13,85].

Overall, water provision can increase predator impacts on prey populations [3,10,15–17], favor species more dependent on water [4,5,65], alter species distributions on the landscape [3–5,10,40,67], reduce the abundance of rarer species [5,10,65], foster range degradation [13,14,81, 83–85], decrease ecosystem stability and induce biodiversity loss [3,5,10,12–14, 65]. Paradoxically, these outcomes counter justification for water establishment. So why do many areas continue to provide water for wildlife, especially year-round? One answer is a credulous belief that managed waters benefit wildlife, without consequences. A second answer is economical, with year-round water provisioning being the simplest and cheapest method of managing water for wildlife. Additional costs (time and money) are required to alter the timing of water provisioning throughout the year, and some waters would be difficult to modify. As a result, we often accept the low economic costs while failing to consider the biological ones.

Wildlife, water, and weather are inextricably linked in arid ecosystems. Our efforts centered on unraveling these connections. By doing so, we offered a straightforward approach to examine factors influencing why and when mammals visited water. The results inform management decisions and techniques regarding the provision of water for mammals, while revealing the biological interaction between predators and prey at water sources.

## Supporting Information

S1 Dataset Project Dataset. Excel spreadsheets with visitation, weather and prey data used in analyses.
(XLSX)

S1 Table Trail Camera Summary. The elevation, start date, end date and duration (days) of trail cameras operating at water sources in Sevilleta National Wildlife Refuge, New Mexico, USA.
(PDF)

S2 Table Correlation. Pearson’s Correlation between variables describing weather and time for predicting visitation to water for 9 mammals at Sevilleta NWR, New Mexico, USA (BC = bobcat, CT = cottontail rabbit, JR = jackrabbit, PH = pronghorn, MD = mule deer, CY = coyote).
(PDF)

S3 Table Model Summary. Models within 7.0 ΔAIC of the model receiving most support, for predicting visitation to water for 9 mammals at Sevilleta National Wildife Refuge, New Mexico, USA (MW = model weight).
(PDF)
