# Supplementary material for: Weather and Prey Predict Mammals’ Visitation to Water
Source: PLoS One. 2015 Nov 11;10(11):e0141355. doi: 10.1371/journal.pone.0141355 (PMC4641626; doi:10.1371/journal.pone.0141355)
Supplement: S1 Table — The elevation, start date, end date and duration (days) of trail cameras operating at water sources in Sevilleta National Wildlife Refuge, New Mexico, USA. (PDF) [file pone.0141355.s002.pdf]

S1 Table. The elevation, start date, end date and duration (days) of trail cameras operating at water sources in Sevilleta National Wildlife Refuge, New Mexico, USA.

Camera ID	Elevation	Start Date	End Date	Duration	Water Source[a]
1	1728	7/23/2009	2014/9/13	1879	Managed
2	1573	1/1/2010	2013/8/26	1334	Managed
3	1548	7/24/2009	2014/7/13	1816	Managed
4	1547	7/23/2009	2014/8/9	1844	Managed
5	1544	7/13/2009	2014/9/15	1891	Managed
6	1588	7/13/2009	2014/8/14	1859	Managed
7	1587	7/12/2009	2014/1/7	1641	Managed
8	1694	8/18/2009	2010/8/11	359	Natural
9	1694	12/23/2010	2013/6/11	902	Natural
10	1693	1/12/2011	2013/6/11	882	Natural
11	1680	6/3/2010	2013/6/9	1103	Natural
12	1848	6/2/2010	2013/3/8	1011	Natural
13	1470	1/8/2010	2014/8/20	1686	Managed
14	1597	7/13/2009	2014/1/8	1641	Managed
15	1594	7/14/2009	2014/9/15	1890	Managed
16	1830	9/17/2009	2014/6/23	1741	Managed
17	1680	6/29/2009	2014/1/14	1661	Managed
18	1676	6/29/2009	2014/9/4	1894	Managed
19	1640	9/2/2010	2014/1/8	1225	Managed
20	1590	1/19/2011	2012/10/11	632	Managed
21	1652	6/29/2009	2014/9/4	1894	Managed
22	1985	9/16/2009	2014/6/3	1722	Managed
23	1985	8/5/2010	2014/1/14	1259	Managed
24	1678	6/29/2009	2014/1/14	1661	Managed
25	1672	6/29/2009	2014/6/10	1808	Managed
26	1809	9/21/2009	2014/9/4	1810	Managed
27	1806	8/7/2010	2014/9/12	1498	Managed
28	1612	5/25/2011	2014/5/12	1084	Natural
29	1660	12/20/2010	2014/3/23	1190	Natural
30	1801	1/13/2011	2014/5/20	1224	Managed
31	1800	1/13/2011	2014/1/14	1098	Managed
32	1469	7/4/2009	2014/1/14	1656	Managed
33	1681	5/22/2010	2014/2/6	1357	Managed
34	1685	6/30/2009	2014/9/4	1893	Managed
35	1759	7/23/2009	2014/8/13	1848	Managed
36	1759	7/23/2009	2014/8/18	1853	Managed

[a] Managed refers to a water source supplied by humans, be it a tank, trough, borehole etc. Natural includes water that comes from seeps, springs, or tinajas, that may be impounded, but not filled by humans via vehicle, or pumps etc.
