# Supplementary material for: Weather and Prey Predict Mammals’ Visitation to Water
Source: PLoS One. 2015 Nov 11;10(11):e0141355. doi: 10.1371/journal.pone.0141355 (PMC4641626; doi:10.1371/journal.pone.0141355)
Supplement: S2 Table — Pearson’s Correlation between variables describing weather and time for predicting visitation to water for 9 mammals at Sevilleta NWR, New Mexico, USA (BC = bobcat, CT = cottontail rabbit, JR = jackrabbit, PH = pronghorn, MD = mule deer, CY = coyote). (PDF) [file pone.0141355.s003.pdf]

S2 Table. Pearson's Correlation between variables describing weather and time for predicting visitation to water for 9 mammals at Sevilleta NWR, New Mexico, USA (BC = bobcat, CT = cottontail rabbit, JR = jackrabbit, PH= pronghorn, MD = mule deer, CY = coyote).

| | Week[a] | TMAX[b] | RH[c] | VPD[d] | P1[e] | P6[f] | BC | Elk | Oryx | Puma | CT | JR | PH | MD | CY |
|---|---|---|---|---|---|---|---|---|---|---|---|---|---|---|---|
| Week | 1.00 | 0.08 | 0.26 | -0.13 | 0.16 | 0.30 | -0.07 | -0.02 | -0.18 | -0.01 | -0.15 | -0.26 | -0.07 | 0.04 | 0.02 |
| TMAX | 0.08 | 1.00 | -0.54 | 0.68 | 0.16 | 0.25 | 0.51 | 0.28 | 0.10 | 0.01 | 0.04 | 0.01 | 0.36 | 0.16 | -0.01 |
| RH | 0.26 | -0.54 | 1.00 | -0.71 | 0.41 | 0.35 | -0.49 | -0.36 | -0.31 | -0.22 | -0.25 | -0.28 | -0.49 | -0.46 | -0.39 |
| VPD | -0.13 | 0.68 | -0.71 | 1.00 | -0.21 | -0.28 | 0.69 | 0.38 | 0.41 | 0.23 | 0.35 | 0.24 | 0.57 | 0.45 | 0.30 |
| P1 | 0.16 | 0.16 | 0.41 | -0.21 | 1.00 | 0.47 | -0.09 | -0.15 | -0.22 | -0.11 | -0.18 | -0.17 | -0.15 | -0.25 | -0.34 |
| P6 | 0.30 | 0.25 | 0.35 | -0.28 | 0.47 | 1.00 | -0.25 | -0.38 | -0.45 | -0.32 | -0.44 | -0.36 | -0.48 | -0.56 | -0.40 |
| BC | -0.07 | 0.51 | -0.49 | 0.69 | -0.09 | -0.25 | 1.00 | 0.32 | 0.40 | 0.20 | 0.36 | 0.24 | 0.53 | 0.47 | 0.39 |
| Elk | -0.02 | 0.28 | -0.36 | 0.38 | -0.15 | -0.38 | 0.32 | 1.00 | 0.41 | 0.37 | 0.26 | 0.14 | 0.55 | 0.57 | 0.25 |
| Oryx | -0.18 | 0.10 | -0.31 | 0.41 | -0.22 | -0.45 | 0.40 | 0.41 | 1.00 | 0.44 | 0.29 | 0.39 | 0.58 | 0.56 | 0.35 |
| Puma | -0.01 | 0.01 | -0.22 | 0.23 | -0.11 | -0.32 | 0.20 | 0.37 | 0.44 | 1.00 | 0.20 | 0.36 | 0.33 | 0.40 | 0.23 |
| CT | -0.15 | 0.04 | -0.25 | 0.35 | -0.18 | -0.44 | 0.36 | 0.26 | 0.29 | 0.20 | 1.00 | 0.38 | 0.55 | 0.53 | 0.33 |
| JR | -0.26 | 0.01 | -0.28 | 0.24 | -0.17 | -0.36 | 0.24 | 0.14 | 0.39 | 0.36 | 0.38 | 1.00 | 0.33 | 0.21 | 0.19 |
| PH | -0.07 | 0.36 | -0.49 | 0.57 | -0.15 | -0.48 | 0.53 | 0.55 | 0.58 | 0.33 | 0.55 | 0.33 | 1.00 | 0.80 | 0.43 |
| MD | 0.04 | 0.16 | -0.46 | 0.45 | -0.25 | -0.56 | 0.47 | 0.57 | 0.56 | 0.40 | 0.53 | 0.21 | 0.80 | 1.00 | 0.62 |
| CY | 0.02 | -0.01 | -0.39 | 0.30 | -0.34 | -0.40 | 0.39 | 0.25 | 0.35 | 0.23 | 0.33 | 0.19 | 0.43 | 0.62 | 1.00 |

[a] Julian week of the year (values spanning 1 – 52).
[b] Weekly average of daily maximum temperature.
[c] Weekly average of daily minimum relative humidity.
[d] Weekly average of daily maximum vapor pressure deficit.
[e] Weekly sum of precipitation.
[f] Sum of precipitation over the prior 5 week period (6-week total amount of precipitation).
