# Supplementary material for: Weather and Prey Predict Mammals’ Visitation to Water
Source: PLoS One. 2015 Nov 11;10(11):e0141355. doi: 10.1371/journal.pone.0141355 (PMC4641626; doi:10.1371/journal.pone.0141355)
Supplement: S3 Table — Models within 7.0 ΔAIC of the model receiving most support, for predicting visitation to water for 9 mammals at Sevilleta National Wildife Refuge, New Mexico, USA (MW = model weight). (PDF) [file pone.0141355.s004.pdf]

S3 Table. Models within 7.0 ΔAIC of the model receiving most support, for predicting visitation to water for 9 mammals at Sevilleta National Wildife Refuge, New Mexico, USA.

Species	Covariates					# Parameters[a]	AIC	ΔAIC	AIC Weight
Cottontail rabbit	VPD		P6			4	1411.9	0.0	0.69
	TMAX		P6			4	1414.9	3.0	0.15
			P6			3	1416.1	4.2	0.08
		RH	P6	RH:P6		5	1416.5	4.6	0.07
Elk	TMAX		P6	TMAX:P6		5	2096.0	0.0	1.00
Jackrabbit		RH	P6	RH:P6		5	1642.1	0.0	0.35
		RH	P6			4	1642.4	0.3	0.30
	VPD		P6			4	1643.2	1.1	0.20
			P6			3	1643.7	1.6	0.16
Mule deer	TMAX	RH	P6	TMAX:P6		6	2976.0	0.0	0.73
	TMAX	RH	P6	TMAX:RH		6	2979.0	3.0	0.16
	TMAX	RH	P6			5	2981.3	5.3	0.05
	TMAX	RH	P6	TMAX:P1	P1	7	2981.9	5.9	0.04
	TMAX		P6	TMAX:P6		5	2982.9	6.9	0.02
Oryx	VPD		P6	VPD:P6		5	1844.0	0.0	0.51
	VPD		P6			4	1845.0	1.0	0.31
		RH	P6	RH:P6		5	1846.8	2.8	0.13
	TMAX	RH	P6	TMAX:RH		6	1849.9	5.9	0.03
	TMAX		P6			4	1850.6	6.6	0.02
Pronghorn	TMAX		P6	TMAX:P6		5	2668.8	0.0	1.00
Bobcat	TMAX	RH	P6	TMAX:P6	Lagomorphs[b]	7	835.0	0.0	0.22
	TMAX	RH	P6	TMAX:P6	CottonTail	7	835.5	0.5	0.17
	TMAX	RH	P6	TMAX:P6		6	835.9	0.9	0.14
	TMAX	RH	P6		Lagomorphs	6	836.4	1.4	0.11
	TMAX	RH	P6		CottonTail	6	837.0	2.0	0.08
	TMAX	RH	P6			5	837.5	2.5	0.06
	TMAX	RH	P6		Jackrabbit	6	837.5	2.5	0.06

	TMAX	RH			Lagomorphs		5	839.0	4.0	0.03
	TMAX		P6		Lagomorphs		5	839.5	4.5	0.02
	TMAX	RH		TMAX:RH	CottonTail		6	840.2	5.2	0.02
	TMAX	RH			CottonTail		5	840.4	5.4	0.01
	TMAX		P6		Jackrabbit		5	840.4	5.4	0.01
	VPD		P6	Week	Week^2		6	840.5	5.5	0.01
	TMAX		P6		CottonTail		5	840.6	5.6	0.01
	TMAX		P6				4	840.9	5.9	0.01
	TMAX	RH			Jackrabbit		5	841.8	6.8	0.01
Coyote	TMAX	RH	P6	P1:P6	P1	Muledeer	8	2036.0	0.0	0.55
	TMAX	RH	P6		P1	Muledeer	7	2037.8	1.8	0.22
	TMAX	RH			P1	Muledeer	6	2039.9	3.9	0.08
	TMAX	RH	P6	TMAX:RH		Muledeer	7	2040.0	4.0	0.07
	TMAX	RH	P6			Muledeer	6	2041.3	5.3	0.04
	TMAX	RH				Muledeer	5	2041.9	5.9	0.03
Puma	Elk		Oryx			Muledeer	5	914.8	0.0	0.49
	Elk				Oryx		4	915.2	0.4	0.40
					Oryx	Muledeer	4	917.7	2.9	0.11

[a] Number of parameters includes the intercept and dispersion parameter.

[b] Lagomorphs consists of cottontail and jackrabbit visitation combined.
